# Differential contributions of working memory and inhibitory control to prospective memory detection and retrieval

**DOI:** 10.3389/fpsyg.2026.1871302

**Published:** 2026-08-04

**Authors:** Sumyah A. Alnajashi

**Affiliations:** Department of Psychology, College of Education, King Saud University, Riyadh, Saudi Arabia

**Keywords:** action video games, cue detection, executive function, inhibitory control, intention retrieval, prospective memory, working memory

## Abstract

**Background:**

Prospective memory (PM)—remembering to carry out intended actions at the right moment — depends on executive control, but the specific contributions of working memory and inhibitory control to PM cue detection versus intention retrieval remain debated.

**Methods:**

Eighty-four adults (44 action video game players, 40 non-players) aged 18–45 completed a within-subjects PM paradigm with separate detection and retrieval conditions, along with measures of working memory (digit span forward/backward, Corsi block), inhibitory control (Stroop interference), and nonverbal reasoning (Raven’s Colored Progressive Matrices). Multiple regression and a stacked long-format model with cluster-robust standard errors tested predictor-outcome associations and their interaction with PM component.

**Results:**

Backward digit span (executive working memory) was a significant positive predictor of PM detection (*β* = 0.32, *p* = 0.009) but not PM retrieval; follow-up analyses confirmed the effect was carried by executive manipulation rather than verbal storage. Stroop interference was the only significant predictor of PM retrieval (*β* = −0.25, *p* = 0.042). The PM-component × working-memory interaction was significant (*β* = −0.34, *p* = 0.009), indicating that working memory was more strongly associated with detection than with retrieval, whereas the corresponding interaction for Stroop interference was not (*β* = −0.06, *p* = 0.649). Action video game experience was not associated with performance on any measure.

**Conclusion:**

Working memory was selectively associated with the detection phase of PM, providing partial support for multiprocess accounts in which different executive mechanisms are differentially recruited across PM phases. The role of inhibitory control was less phase-specific. Modest action video game experience does not confer measurable PM advantages.

## 1 Introduction

Remembering to take medication, return a phone call, or stop at a store on the way home all depend on prospective memory (PM), the ability to encode an intention now and execute it later, when an appropriate cue or moment occurs (Einstein and McDaniel, 1990; McDaniel and Einstein, 2007). PM is not a unitary capacity. Successful prospective remembering requires at least two dissociable component demands: detecting that the moment for action has arrived, and retrieving the content of the intended action and executing it while the ongoing task pulls attention in another direction (Ball et al., 2022; Einstein et al., 2005; Scullin et al., 2017). These two demands plausibly recruit different executive resources, but the literature has rarely separated them within a single paradigm and tested whether their executive predictors dissociate. The present study takes that as its central question. Despite the centrality of PM to independent everyday functioning, it has received less empirical attention than retrospective memory (Bediou et al., 2018; Guzzardi et al., 2024).

Contemporary theoretical accounts specify when PM relies heavily on strategic monitoring, which requires controlled attention (e.g., action is required after certain time), and spontaneous retrieval, which is based on cues or events (e.g., when the alarm rings). Preparatory attention and memory processes (PAM) theory proposes that successful event-based PM requires alertness and controlled attention and retrospective memory (Smith, 2003; Smith and Bayen, 2005). In contrast, multiprocess and dynamic frameworks propose that PM can be supported by either strategic monitoring or spontaneous retrieval, depending on features such as cue salience and ongoing task demands (Einstein et al., 2005). These theoretical perspectives align around the notion that prospective memory functioning depends on executive control mechanisms that facilitate maintaining goals, directing attention, managing interference, and revising information (Burgess et al., 2011; Cona et al., 2015).

### 1.1 Prospective memory components and cognitive control

Prospective memory depends on several processes that are frequently discussed in the literature, including inhibitory control (resisting distraction from the ongoing task), updating (keeping the intention active while other information changes), and retrospective memory (remembering the content of the intention; Ball et al., 2022; Ballhausen et al., 2017).

PM components are examined in lab experiments by integrating ongoing tasks with two distinct types of PM tasks (Ball et al., 2022; Laera et al., 2021). A study by Ball et al. (2022) used PM tasks that varied in detection vs. retrieval demands to isolate PM components. The detection task demands attention to detect a less salient cue with minimal retrieval demands (e.g., pressing a specific key when words are semantically related). In contrast, the retrieval task demands retrieval of the content of intention and related actions based on salient cues (e.g., press one of two keys based on the number of syllables when words meet a certain condition).

Working memory and inhibitory control have both been implicated in prospective memory, but the literature suggests they may contribute differentially across PM phases. Working memory capacity supports the active maintenance of intentions and the controlled allocation of attention to monitor for cues, processes that are particularly demanding when cues are non-focal or embedded in an attentionally demanding ongoing task (Smith and Bayen, 2005; Unsworth and Engle, 2007). Individuals with greater working memory capacity show better goal-maintenance and reduced goal-neglect under interference (Kane and Engle, 2003), suggesting that working memory should be most consequential during the cue-detection phase, when the intention must be held in an accessible state and matched against incoming stimuli. Inhibitory control, in contrast, is theoretically more central to the retrieval phase. Once a cue has been detected, the participant must suppress the prepotent ongoing-task response and switch to executing the intention (Bugg and Ball, 2017). Stroop-type interference indices are therefore predicted to relate more strongly to PM retrieval than to PM detection. To date, no prior study has directly tested this differential pattern within a single paradigm that separates detection and retrieval demands, leaving the question of whether working memory and inhibitory control make distinct or overlapping contributions to PM components empirically open.

Recent meta-analytic and neuroimaging work has begun to localize these component demands to partially distinct mechanisms. The Attention-to-Delayed-Intentions model (Cona et al., 2015) and subsequent work (Cantarella et al., 2023) propose a dorsal/ventral functional dissociation: dorsal frontoparietal regions support top-down monitoring during the cue-detection phase, while ventral parietal regions are recruited when a cue is detected and the intended action must be retrieved and executed. A meta-analysis of strategic monitoring (Peper and Hunter Ball, 2023) shows that contextual cueing improves PM specifically by reducing monitoring costs, consistent with the view that the detection phase is the working-memory–limited bottleneck. At retrieval, by contrast, mouse-tracking and ERP evidence (Kurtz et al., 2022; Yu et al., 2023) implicate inhibitory mechanisms in the suppression of the ongoing-task response and in the deactivation of the intention once executed. Convergent evidence thus predicts that working memory should track PM detection more closely than PM retrieval, while a Stroop-type interference index—which captures the efficiency of suppressing a prepotent response—may track PM retrieval more closely than detection.

### 1.2 PM and action video games

A secondary question concerns whether everyday cognitive habits—specifically, action video game experience—predict PM performance over and above standard executive-function measures. Action video games place sustained demands on selective attention, rapid target detection, and inhibition of distractors under time pressure (Bavelier et al., 2012; Green and Bavelier, 2003), and cross-sectional meta-analyses have linked action video game play to advantages on attentional and perceptual tasks (Bediou et al., 2018; Powers et al., 2013), with more recent work suggesting that the broadest transfer is to learning rate rather than to specific higher-order capacities (Bavelier and Green, 2025; Smith and Basak, 2023). At the same time, transfer to executive functions and to memory is small, inconsistent, and dependent on stimulus and response overlap between game and task (Boot et al., 2011; Sala et al., 2018). In the present study, action video games refer to fast-paced games requiring continuous real-time control, rapid decisions, frequent target detection, and high attentional load, typically including first- or third-person shooters and action combat games (Green and Bavelier, 2003; Boot et al., 2011; Bediou et al., 2018). These demands map onto PM component processes, especially those emphasized by PAM and multiprocess models (Smith, 2003; McDaniel and Einstein, 2007). Whether action video game experience is associated with prospective remembering, and whether any such association is greater for the more attentionally demanding detection phase than for the retrieval phase, has not been examined directly.

### 1.3 The present study

The present study had two aims. First, and primarily, the study tested whether working memory and inhibitory control differentially predict PM detection and PM retrieval. Drawing on the multiprocess and Attention-to-Delayed-Intentions frameworks (Cantarella et al., 2023; Cona et al., 2015; Einstein et al., 2005; McDaniel and Einstein, 2007), I hypothesized (H1) that working memory capacity would more strongly predict PM detection—the phase that requires sustained monitoring for non-focal cues—and (H2) that inhibitory control, indexed by Stroop interference, would more strongly predict PM retrieval, the phase that requires suppressing the ongoing-task response and switching to the intention-related response. The secondary, exploratory aim was to examine whether action video game experience was associated with PM performance and with the executive functions that support it. Given that action gaming most reliably sharpens rapid attentional capture and target detection, it was further predicted (H3) that any gaming-related advantage in PM would be larger for the detection phase than for the retrieval phase.

The study also included a measure of nonverbal reasoning (Raven’s Colored Progressive Matrices) as a control variable to evaluate whether any observed group differences are specific to executive control rather than reflecting broader differences in general cognitive ability.

## 2 Materials and methods

### 2.1 Participants

Eighty-four adults (*M* = 24.44 years, SD = 6.56, range 18–45; 71 women, 13 men) were recruited in Saudi Arabia through the university social network and announcements in social media. The marked predominance of women reflects convenience recruitment from the psychology department and university social network in which women are over-represented rather than a deliberate sampling decision; the resulting gender imbalance is considered in the Limitations. Some participants were from the psychology department and received course credits in return for their participation. All participants were native Arabic speakers. Most of the participants were from middle socio-economic status (77.38, 14.47% from high and 3.57% from low socio-economic status). University students were 76.19, 11.9% were employed, 8.33% were not employed, and the rest did not report their status. Age differed between action video game players (*n* = 44) and non-players (*n* = 40), Welch’s *t*(51.37) = 5.01, *p* < 0.001 (non-players: *M* = 27.83, SD = 7.60; players: *M* = 21.34, SD = 3.20). Therefore, age was included as a covariate in subsequent regression analyses. Gender was not entered into the primary models because the marked imbalance (71 women, 13 men) would yield an unstable estimate based on only 13 men; its effect was instead examined in a sensitivity analysis (Supplementary Table S3). Forty-eight participants completed the study in the psychology testing laboratory, whereas 36 participants completed it online using their devices. Data-collection mode (online vs. in person) was recorded for each participant and was included as a factor in main analyses. The study was approved by the Research Ethics Committee of King Saud University, and all participants provided written informed consent. A sensitivity analysis in G*Power 3.1 (Faul et al., 2007) *α* = 0.05, power = 0.80 indicated that, at the analytic sample size for models containing the Stroop measure (*N* = 70), the smallest detectable effect was *f*^2^ = 0.12 for an individual predictor and *f*^2^ = 0.18 for the full four-predictor model.

### 2.2 Instruments

#### 2.2.1 Prospective memory task

The present study employed a within-subject prospective memory (PM) paradigm with three blocks: (a) ongoing task performance, (b) PM detection, and (c) PM retrieval. All experimental programming was carried out in PsychoPy (Peirce et al., 2019) on a Windows laptop at 1920 × 1,080 resolution with a 60 Hz refresh rate. All verbal stimuli were presented in Arabic, the participants’ native language. On each trial, the two words were displayed side by side in white on a black background in 24 pt. The word pool consisted of 100 concrete, picturable Arabic nouns selected from the normed object-word set of Albalawi and Alnajashi (2022), in which each item had been rated by independent samples for Arabic word frequency and familiarity and documented for letter length. Only high-frequency, early-acquired items were retained, so that all words were common and easily recognized. On each trial, two of these nouns were presented side by side. Individual words were 2–6 letters (*M* = 4.1), and the displayed word pair ranged from 6 to 12 letters in total (*M* = 8.4). Each noun recurred across approximately eight pairs, with pairing and left/right position varied, yielding 400 word-pair trials across the ongoing (80), detection (160), and retrieval (160) blocks. The overall design was adapted from Ball et al. (2022), itself based on the framework introduced by Simons et al. (2006). Throughout, the labels “detection” and “retrieval” denote the dominant processing demand of each block rather than process-pure measures of a single PM component.

In the ongoing block, participants viewed pairs of unrelated words and pressed “F” or “J” to indicate which word contained more letters. The detection block used the same ongoing task, but participants were instructed in advance that whenever both words happened to begin with the same letter—the PM cue—they should withhold the F/J response and instead press “A.” This block isolates the detection demand of PM: the cue is a non-salient lexical feature embedded in the ongoing task, but the response, once the cue is noticed, is minimal.

In the retrieval block, the ongoing task was again the F/J letter-comparison task, but on PM-cue trials the word pair appeared in Andalus font (visually distinctive against the default sans-serif font of the ongoing trials). When this cue was present, participants were to apply a previously learned rule: count the total number of letters across both words and press “L” if the total exceeded eight, otherwise “S.” The cue is therefore highly salient (the font change is automatically detected), but the response requires holding the multi-step retrieval rule in memory and executing it correctly. This block isolates the retrieval-and-execution demand of PM with detection demand minimized.

Each word-pair stimulus remained on screen until the participant responded, up to a maximum of 3 s; the response terminated the display and the next trial followed immediately, with no inter-trial interval and no trial-by-trial feedback during the experimental blocks. Within each block, PM-cue trials were interspersed among ongoing-task trials at intervals of 4–10 trials, following the spacing reported by Laera et al. (2021), in a fixed sequence identical across participants. Each block yielded 24 p.m.-cue trials, and the whole task took up to 10 min. The primary dependent measure was accuracy (percent correct) on PM-cue trials in the detection and retrieval blocks. Internal consistency of the PM-accuracy measures (each cue trial treated as a dichotomous item) was good, KR-20 = 0.89 for detection and 0.83 for retrieval.

#### 2.2.2 Raven’s progressive matrices

Raven’s Colored Progressive Matrices were used to assess general nonverbal intelligence (Ali, 2013). This Arabic validated version contains 36 items, and participants were allotted 6 min to complete as many matrices as possible. Performance was quantified by the total number of correctly solved items. Reliability for the population, as calculated by the Kuder–Richardson formula, was 0.92. It was administered as a timed online form built in Google Forms. Because the matrices served only as a control covariate for general nonverbal ability rather than as a focal measure, online administration was considered acceptable.

#### 2.2.3 Stroop task

The Stroop Color and Word Test assesses behavioral inhibition by having participants identify the ink color of color-denoting words that appear in either matching or conflicting hues. Participants saw four Arabic color words (the equivalents of red, blue, green, and yellow), displayed one at a time in either matching (congruent) or non-matching (incongruent) ink colors. Each trial began with a 500 ms fixation cross, followed immediately by the word stimulus, which remained on screen until the participant pressed one of four response keys corresponding to ink color. Reaction times and accuracy were recorded, and an interference score was calculated as the difference between mean response time on incongruent versus congruent trials. The task was built with PsychoPy (Peirce et al., 2019), and responses were given by pressing assigned buttons on the keyboard.

#### 2.2.4 Computer-based working-memory tasks

A digit-span paradigm (forward and backward recall) was adapted from the Arabic version of the Wechsler Adult Intelligence Scale Revised (Melika, 1996), and a Corsi-block–style grid task for visuospatial memory, using forward span only (Corsi, 1972; Kessels et al., 2000). In both tasks, sequence length was incrementally increased, and two consecutive failures at a given length defined the participant’s span, following the standardized procedure described by Kessels et al. (2000). The task was built in React, using JavaScript.

#### 2.2.5 Study questionnaire

Video game experience was assessed with a brief self-report questionnaire delivered in Google Forms alongside the consent form. Participants reported (a) whether they currently played video games, (b) the titles and genres they played most often, (c) the typical number of play sessions per week, and (d) the typical duration of a session. Game classification was based on participants’ self-report; titles named by each participant were checked against the action criteria by the author, but play frequency and duration were not independently verified, a limitation noted in the Limitations section. Following established definitions in the action-video-game literature (Green and Bavelier, 2003; Bediou et al., 2018), action games were operationalized as fast-paced titles placing continuous demands on rapid target detection, divided attention, and time-pressured responding, principally first- and third-person shooters (e.g., Call of Duty, PUBG Mobile, Free Fire, Fortnite). Titles lacking these demands, for example, casual, puzzle, simulation, and dress-up/fashion games (e.g., The Sims), were classified as non-action. Participants were classified as action video game players if they reported regular play (averaging ≥ 30 min/day, derived from the reported number of weekly sessions and session duration, over the preceding 6 months) of one or more games meeting the action criterion, and as non-players otherwise; players of exclusively non-action genres and infrequent players of action titles therefore fell into the non-player group. This procedure yielded 44 action video game players and 40 non-players.

### 2.3 Procedures

**Figure 1:**
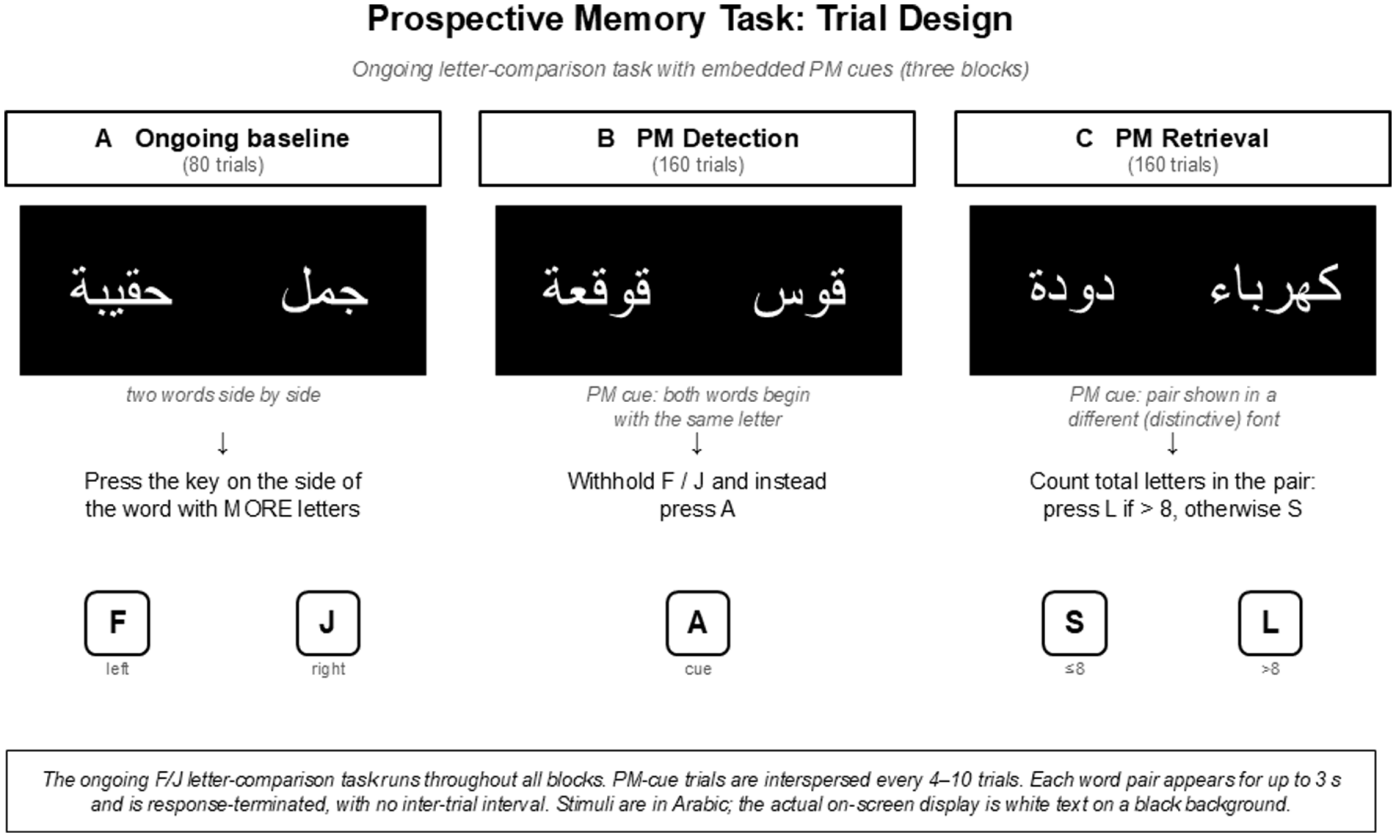
Schematic of the prospective memory (PM) task design. There were three blocks: **(A)** Ongoing-baseline block (80 trials), **(B)** The PM detection block (160 trials), and **(C)** The PM retrieval block (160 trials).

Upon completion of the online questionnaire, participants were contacted via email to schedule a visit to the psychology lab in the College of Education at King Saud University to complete the gaming experiment or book an online slot. Participants were tested individually. After providing informed consent, participants were randomly assigned to complete a 15-min pre-task activity, either a car racing game or a Sudoku puzzle, to standardize arousal and engagement across participants prior to testing. Participants then received standardized instructions and completed a series of practice trials for the ongoing task. Once participants demonstrated adequate understanding (at least 80% accuracy in practice), the PM intention was introduced verbally and reinforced with on-screen examples. After this, participants started the main experimental blocks. The session comprised three blocks: (a) ongoing task with 80 trials, (b) prospective memory detection task with 160 trials, and (c) prospective memory retrieval task with 160 trials. Throughout each block, participants kept their hands in a fixed position on the keyboard: in the ongoing-baseline conditions, the index fingers rested on the F and J keys, whereas in the remaining conditions the left little finger rested on the A key and the left and right ring fingers rested on the S and L keys, respectively. See Figure 1 for the task design. The prospective-memory blocks were administered in a fixed order, and no feedback was provided during the experimental blocks. PM demands were blocked rather than intermixed because each required a distinct intention and response rule, so interleaving them would have confounded PM-component demand with continual task-set switching. This was followed by the remaining cognitive tests in counterbalanced orders across participants.

Subsequently, participants completed the battery of cognitive tasks. The whole session lasted up to 60 min. Finally, the participants were debriefed about the study and their questions were answered. The experimental procedure was approved by Institutional Review Board in King Saud University, and the study was conducted in accordance with the Declaration of Helsinki.

### 2.4 Data analysis

Analyses were conducted in R (version 4.3) using the sandwich and lmtest packages for cluster-robust standard errors. Age was included as a covariate in all regression models because it differed significantly between groups; gender was not included in the primary models because of the strong gender imbalance. Data-collection mode was retained as a control in preliminary models to rule out measurement heterogeneity.

Working memory was indexed by backward digit span, which requires active maintenance and manipulation and most directly reflects the executive component implicated in monitoring for non-focal cues. This single-task index was adopted in preference to a composite because the prospective-memory task was verbal, the verbal and visuospatial span measures were weakly and inconsistently correlated, and forward and backward span entered separately showed that only backward span was associated with the outcomes (Supplementary Table S1). A verbal composite (mean of z-standardized forward and backward span) yielded equivalent results (Supplementary Table S6). Stroop interference was indexed as the incongruent–congruent difference in mean response time, with higher scores indicating poorer inhibitory control.

The primary analyses comprised two parallel OLS regressions predicting PM detection and PM retrieval from Stroop interference, working memory, age, and group, entered simultaneously to estimate the unique contribution of each predictor. To formally test whether predictor contributions differed across PM components, scores were stacked into long format and analyzed in a single OLS model with PM component and its interactions with the working memory (backward digit span) and Stroop interference. Cluster-robust standard errors (clustered by participant) corrected for within-person dependence; this was preferred over a mixed model because the dependence structure is simple (two observations per participant) and the focal hypothesis concerns fixed-effect interactions rather than variance components. The Holm–Bonferroni correction was applied to the family of group-effect tests.

Standardized coefficients (*β*), 95% CIs, and semi-partial correlations (sr) are reported as effect sizes; model-level effect sizes are reported as adjusted R^2^. Statistical significance was set at *α* = 0.05 (two-tailed).

## 3 Results

Scores for prospective memory task, Stroop task, and Raven’s Progressive Matrices test were converted to percentages. Table 1 shows descriptive statistics for all cognitive measures by group (action video game players vs. non-players). For the Stroop interference index, higher values indicate greater interference. Correlations among the working memory measures were examined using Pearson correlations. Forward and backward digit span correlated only weakly with visuospatial span, with one of the two correlations reaching significance (forward × Corsi: r = 0.06, *p* = 0.567; backward × Corsi: r = 0.23, *p* = 0.035).

Seven participants were excluded from analyses involving prospective memory, and 14 participants were excluded from analyses involving the Stroop task. Exclusions were due to technical issues, resulting from internet instability during online testing, which led to incomplete or invalid responses. These cases showed unusually poor performance consistent with self-reported connectivity issues. Raw logs revealed pervasive non-responses and atypical key registration (e.g., bracket characters instead of valid responses), with performance at or below chance level across conditions (50% for two alternative tasks and 25% for four-alternative tasks). For the Stroop task, exclusions additionally reflected problems with pressing the required response keys.

**Table 1:** Descriptive statistics for cognitive measures by group (action video game players vs. non-players).

Task	Condition/index	Group	*N*	M	SD	Skewness	Kurtosis
PM	Ongoing	Players	39	87.43	9.21	−2.32	7.56
		Non-players	38	86.63	7.46	−0.88	0.24
	Detection cost	Players	39	86.97	11.07	−1.69	2.09
		Non-players	38	87.94	9.51	−1.46	1.53
	Retrieval cost	Players	39	83.45	16.11	−2.13	4.47
		Non-players	38	82.70	11.18	−1.05	0.24
	PM detection	Players	39	65.06	21.94	−0.64	−0.38
		Non-players	38	61.73	20.55	−0.55	0.16
	PM retrieval	Players	39	53.73	18.11	−0.96	0.72
		Non-players	38	58.38	17.56	−1.17	2.49
Stroop	Congruent ACC	Players	35	87.50	18.58	−2.16	3.62
		Non-players	35	82.79	18.33	−2.13	4.48
	Incongruent ACC	Players	35	83.89	20.36	−2.19	3.87
		Non-players	35	79.35	22.66	−2.04	4.14
	Inhibitory control	Players	35	3.27	5.04	−0.05	0.87
		Non-players	35	3.71	6.40	−0.93	0.98
Raven’s Matrices		Players	43	87.34	9.70	−1.48	2.91
		Non-players	38	86.55	8.51	−0.79	0.73
Working memory	Digit span forward	Players	44	5.45	1.07	0.91	0.45
		Non-players	39	5.54	0.99	0.23	0.47
	Digit span backward	Players	44	4.66	1.09	−0.48	−0.20
		Non-players	39	4.33	0.84	0.14	−0.44
	Visuospatial WM	Players	44	5.20	1.61	−0.53	−0.33
		Non-players	39	5.08	1.38	−0.21	−0.89

Additional missing data resulted from task noncompletion and isolated software failure. Raven’s Progressive Matrices data were missing for (1/44, 2.3%) of action video game players and (2/40, 5%) of non-players; no imputation was applied because this task was used to compare groups in general cognitive ability, and the amount of missingness was small. One participant did not complete the working memory tasks, and primary regression analyses used complete observations across variables. Two isolated PM-condition scores were missing due to software failure in saving the output file (one in the ongoing PM condition, and one in the PM retrieval condition). As each failure affected only a single condition score, these values were replaced with the sample mean for the corresponding condition. Sensitivity analyses re-estimating the primary models while leaving these cells missing showed no change in statistical conclusions.

Missing data patterns were evaluated to assess whether data loss was systematic. For prospective memory performance, between-group differences and missingness were evaluated using Fisher’s exact test due to small expected cell counts. Missingness was not associated with gaming status (*p* = 0.283) or pre-task activity (*p* = 0.851). In contrast, missingness differed by data-collection mode (online vs. in-person), Fisher’s exact *p* = 0.002, with missing data occurring only in the online condition (7/36, 19.4%) and none in the in-person condition (0/48); overall, missingness was 7/84 (8.3%). For Stroop task performance, Pearson’s *χ*^2^ tests were used because expected cell-count assumptions were satisfied. Gaming status and pre-task activity were not significant, Pearson’s *χ*^2^ (1) = 0.15, *p* = 0.696, Pearson’s *χ*^2^(1) = 2.15, *p* = 0.142, respectively, whereas association with data-collection mode was significant Pearson’s *χ*^2^(1) = 17.15, *p* < 0.001. The same mode specific pattern was observed for the Stroop task (online 13/36, 36.10%; in person: 1/48, 2.08%; overall: 14/84, 16.66%). The consistent effects of administration mode suggest that online testing may have been more susceptible to technical disruptions.

With regard to non-verbal intelligence scores, indexed by Raven’s Progressive Matrices, no significant differences were found between gamers (*M* = 87.34, SD = 9.70) and non-gamers (*M* = 86.55, SD = 8.51), *t*(79) = 0.387, *p* = 0.35.

Preliminary analyses using ordinary least squares regression tested the effects of group (players vs. non-players), pre-task activity (car racing vs. Sudoku), and data-collection mode (online vs. in-person) on each outcome. Analyses used listwise deletion for missing data; the final sample was 77 for PM conditions and 70 for Stroop. Pre-task activity did not significantly predict performance on any outcome, and interactions between group and pre-task activity were nonsignificant. Pre-task activity was therefore not retained in subsequent analyses. The group effect was nonsignificant for all outcomes, all Holm-adjusted *p* = 1.000. Data-collection mode was significantly associated with PM cost outcomes (online accuracy lower than in-person), but not with PM detection, PM retrieval, or Stroop interference. Data-collection mode was retained as a covariate in the primary analyses. Table 2 shows regression results for group, pre-task activity, and data-collection mode as predictors of PM outcomes and Stroop interference.

**Table 2:** Regression results: effects of group, pre-task activity, and data-collection mode on PM outcomes and Stroop interference.

Outcome	Group			Pre-task activity		Data-collection mode	
	*B*	*p*	Holm-adj *p*	*B*	*p*	*B*	*p*
Ongoing task	0.42	0.832	1.000	−2.27	0.304	−1.68	0.464
PM detection cost	−1.65	0.475	1.000	−2.14	0.408	−5.83	0.032
PM retrieval cost	−0.47	0.876	1.000	2.37	0.485	−9.80	0.007
PM detection	3.30	0.510	1.000	3.86	0.491	1.57	0.787
PM retrieval	−5.03	0.234	1.000	−4.02	0.393	−0.93	0.849
Stroop interference	−0.11	0.937	1.000	−1.13	0.461	1.61	0.330

*B*, unstandardized regression coefficients. Holm-adjusted *p* values apply to the group effect across outcomes.

Reaction time outcomes showed consistently slower responses in the online condition across all measures (all *p* < 0.001), but no significant group or pre-task activity effects. Accuracy was therefore used as the primary dependent variable for all subsequent analyses.

Primary analyses tested whether prospective memory (PM) components: PM detection and PM retrieval were associated with gaming group (action video game players vs. non-players), inhibitory control, and verbal working memory (backward digit span), with age included as a covariate. Continuous variables were z-standardized prior to analyses.

The regression model for PM detection was marginally significant, *F*(4, 65) = 2.42, *p* = 0.057, accounting for 13.0% of the variance, (*R*^2^ = 0.130, adjusted- *R*^2^ = 0.076). Backward digit span (executive working memory) was a significant positive predictor of PM detection, *β* = 0.32, *t*(65) = 2.70, *p* = 0.009, 95% CI [0.08, 0.56], indicating that better executive working memory was associated with higher PM detection. Stroop interference showed a non-significant negative association with PM detection, *β* = −0.13, *t*(65) = −1.16, *p* = 0.251. Age and group were not significant predictors, both *p* > 0.62. Semi-partial correlations indicated that backward working memory accounted for the largest proportion of unique variance in PM detection (sr = 0.31) relative to inhibitory control (sr = −0.13), group (sr = −0.02), and age (sr = −0.06).

To confirm this reflected executive manipulation rather than verbal storage, working memory was decomposed into a storage component (forward span) and an executive component (backward span residualized on forward); only the executive component predicted detection, *β* = 0.30, *p* = 0.015, whereas storage did not, *β* = 0.16, *p* = 0.200 (Supplementary Tables S1, S2).

The regression model predicting PM retrieval was not significant, *F*(4, 65) = 1.70, *p* = 0.161, accounting for 9.5% of the variance (*R*^2^ = 0.095, adjusted- *R*^2^ = 0.039). Stroop interference was the only significant predictor, *β* = −0.25, *t*(65) = −2.08, *p* = 0.042, 95% CI [−0.48, −0.01], indicating that greater inhibitory interference was associated with lower PM retrieval. Backward working memory did not predict retrieval, *β* = 0.06, *t*(65) = 0.48, *p* = 0.630. Age and group were not significant, both *p* > 0.29. Semi-partial correlations indicated that inhibitory control accounted for the largest proportion of unique variance (sr = −0.25), relative to group (sr = −0.12), backward working memory (sr = 0.06), and age (sr = 0.06). Table 3 shows the results of the two multiple regression models predicting PM detection and PM retrieval.

**Table 3:** Two multiple regression models predicting PM detection and PM retrieval.

Outcome	Predictor	*β*	SE	*T*	*p*	95% CI
PM detection	Stroop interference	−0.13	0.12	−1.16	0.251	[−0.37, 0.10]
	Working memory	0.32	0.12	2.70	0.009	[0.08, 0.56]
	Age	−0.07	0.13	−0.51	0.610	[−0.33, 0.20]
	Group	−0.06	0.27	−0.22	0.828	[−0.59, 0.48]
PM retrieval	Stroop interference	−0.25	0.12	−2.08	0.042	[−0.48, −0.01]
	Working memory	0.06	0.12	0.48	0.630	[−0.18, 0.30]
	Age	0.07	0.14	0.53	0.596	[−0.20, 0.34]
	Group	−0.29	0.27	−1.05	0.296	[−0.84, 0.26]

**Figure 2:**
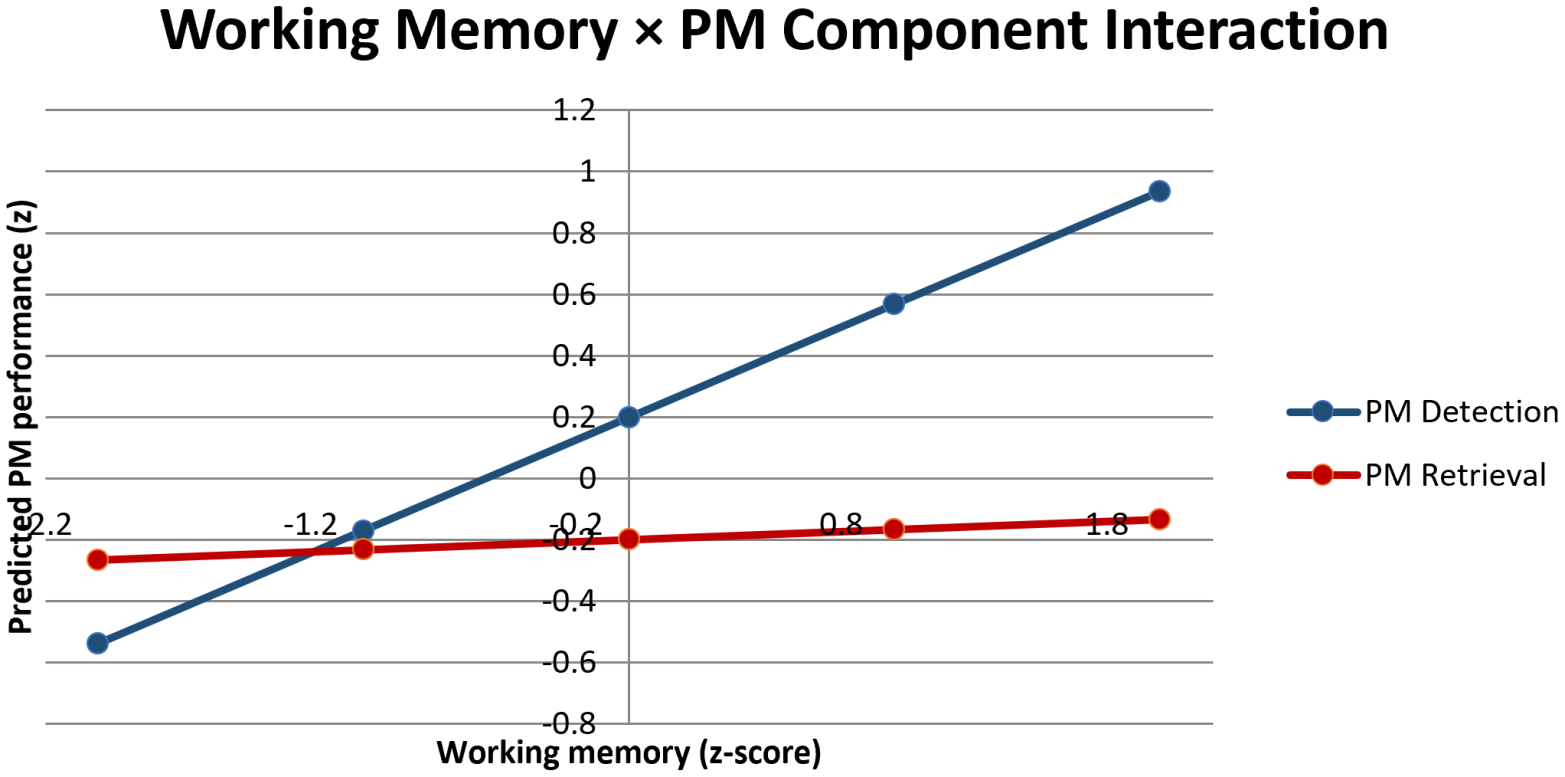
Predicted prospective memory (PM) performance as a function of standardized working memory (backward digit span), shown separately for PM detection and PM retrieval. Values are model-predicted z-scores from the stacked long-format model with cluster-robust standard errors (*N* = 70), with all other predictors held at their sample means. The steeper slope for detection relative to the near-flat slope for retrieval reflects the significant PM component × working memory interaction (*β* = −0.34, *p* = 0.009).

To test whether predictor-outcome relations differ across PM components, PM detection and PM retrieval were stacked into a long-format dataset and analyzed using (OLS) regression, including PM detection vs. PM retrieval, predictors, and interactions. Because each participant has two observations, cluster-robust standard errors were computed with clustering by participant. The overall model accounted for 14.2% of the variance (*R*^2^ = 0.142, adjusted- *R^2^* = 0.097). There was a significant main effect of PM component, *β* = −0.40, SE = 0.14, *t*(132) = −2.94, *p* = 0.003, indicating lower retrieval than detection scores. Across outcomes working memory (backward digit span) was positively associated with PM performance, *β* = 0.37, SE = 0.13, *t*(132) = 2.85, *p* = 0.004, whereas Stroop interference was not, *β* = −0.15, SE = 0.12, *t*(132) = −1.23, *p* = 0.219. The PM-component × working-memory interaction was significant, *β* = −0.34, SE = 0.13, *t*(132) = −2.60, *p* = 0.009, indicating that working memory was more strongly associated with detection (*β* = 0.37) than with retrieval (*β* = 0.03). The PM-component × Stroop interaction was not significant, *β* = −0.06, SE = 0.13, *t*(132) = −0.46, *p* = 0.649. Age and group were not significant predictors (*p* ≥ 0.42). Full model coefficients are reported in Table 4, and the working memory × component interaction is illustrated in Figure 2.

**Table 4:** Stacked long-format regression predicting PM performance from PM component, working memory, Stroop interference, age, and group, with cluster-robust standard errors (clustered by participant; *N* = 70).

Term	*β*	SE	*t*	*p*	95% CI
PM component (retrieval vs. detection)	−0.40	0.14	−2.94	0.003	[−0.66, −0.13]
Working memory (backward digit span)	0.37	0.13	2.85	0.004	[0.12, 0.62]
Stroop interference	−0.15	0.12	−1.23	0.219	[−0.39, 0.09]
Age	−0.01	0.09	−0.07	0.940	[−0.19, 0.17]
Group (player vs. non-player)	−0.15	0.19	−0.81	0.419	[−0.53, 0.22]
PM component × working memory	−0.34	0.13	−2.60	0.009	[−0.59, −0.08]
PM component × Stroop	−0.06	0.13	−0.46	0.649	[−0.30, 0.19]

Control analyses supported these associations: they were unchanged after adding gender as a covariate (Supplementary Table S3); the detection–working-memory effect persisted after controlling for block-wise ongoing-task accuracy (*β* = 0.30, *p* = 0.007), although the retrieval–inhibition effect was attenuated to a trend under the same control (*β* = −0.19, *p* = 0.056; Table S4). Regression assumptions were evaluated for both primary models: residuals showed only mild departures from normality (Shapiro–Wilk *p* = 0.015–0.046), consistent with the bounded accuracy scale, while homoscedasticity (Breusch–Pagan *p* ≥ 0.50), linearity (RESET *p* ≥ 0.16), and multicollinearity (max VIF = 1.36) were all satisfied, and the focal effects were unchanged under HC3 robust standard errors and a 5,000-sample bootstrap (Supplementary Table S5).

## 4 Discussion

The present study tested whether working memory and inhibitory control differentially predict prospective memory detection and retrieval. The results provide partial support for this differential pattern. Working memory was a significant positive predictor of PM detection but not PM retrieval, and the formal PM-component × working memory interaction confirmed that the contribution of working memory was stronger for detection than for retrieval. Inhibitory control, indexed by Stroop interference, was a significant negative predictor of PM retrieval in the separate-model analysis but did not show a significant interaction with PM component in the stacked model. The pattern is therefore best described as a single dissociation, with selective involvement of working memory in detection, accompanied by a numerically consistent but statistically non-significant tendency for inhibitory control to track retrieval more than detection.

Although the pattern superficially resembles a double dissociation, the formal interaction tests support only a single dissociation: the PM-component × working-memory interaction was significant (β = −0.34, *p* = 0.009), whereas the PM-component × Stroop interaction was not (β = −0.06, *p* = 0.649). The present data thus establish that working memory is selectively associated with PM detection but do not establish that inhibition is selectively associated with PM retrieval; the inhibition–retrieval link is treated here as a single, suggestive finding rather than as the second arm of a double dissociation. These patterns suggest that participants with stronger working memory capacity were better able to maintain intentions and monitor for detection cues during multitasking (Miyake et al., 2000; Unsworth and Engle, 2007). This selective association observed for working memory aligns with theoretical accounts emphasizing different executive demands across PM components (Ball et al., 2022; Bugg and Ball, 2017).

The differential executive predictors across PM components provide partial support for multiprocess frameworks. The stronger role for working memory in PM detection was specific to its executive-manipulation component (backward span) rather than passive storage (forward span), consistent with models in which monitoring for non-focal cues draws on controlled, capacity-limited maintenance rather than storage per se. This specificity also addresses the concern that a verbal storage measure might inflate the apparent working-memory contribution. The shift toward inhibitory control as a predictor of PM retrieval performance may reflect demands to suppress the ongoing task and switch to intention-related processes when salient cues appear (Einstein et al., 2005). These findings suggest that although overall differences between gaming groups are absent, individual differences in executive functions systematically relate to PM in theoretically predicted ways.

The pattern observed here partially converges with recent neuroimaging and meta-analytic evidence for a functional dissociation across PM phases. Cantarella et al. (2023) reported that monitoring-phase activity is concentrated in dorsal frontoparietal regions associated with working memory and top-down control, whereas detection-phase activity is concentrated in ventral parietal regions linked more closely to response selection and interference resolution. Peper and Hunter Ball’s (2023) meta-analysis of strategic monitoring further shows that cues which reduce monitoring demands selectively benefit PM, again consistent with monitoring being the limited bottleneck of working memory. Behaviorally, Kurtz et al. (2022) used mouse-tracking to show that retrieval-phase performance reflects inhibitory processes governing both ongoing-task suppression and intention deactivation, mirroring the present association between Stroop interference and PM retrieval. Together with the present results, this pattern is consistent with the view that “executive control” is not a single resource that supports PM uniformly, but a set of partially separable mechanisms whose recruitment may vary by phase. The selective association of working memory with detection in the present data provides direct behavioral support for this view, although stronger conclusions about the phase-specificity of inhibitory control will require studies with greater statistical power.

A further interpretive caution concerns the nature of the tasks themselves. Because the PM cues were rare events embedded within a continuous ongoing task, the detection and retrieval blocks can also be characterized in terms of divided attention and task switching rather than prospective memory per se (Koch et al., 2018). On this reading, the present associations may partly reflect the efficiency of monitoring for, and switching to, infrequent target events—processes that overlap with, but are not unique to, PM. The sustained search for cues required here differs from many everyday PM situations, in which an intention is not actively rehearsed across the retention interval. The present data cannot differentiate between a strictly prospective-memory account and a broader attentional-control or multitasking account, and the working-memory and inhibition associations reported here are compatible with both. The findings are therefore best framed as informative about the executive correlates of these PM-analogue tasks rather than as isolating process-pure PM components.

Turning to the secondary question, action video game experience was not associated with PM in either component, nor with the executive measures that predicted PM. This null is consistent with the recent meta-analytic picture, in which transfer from action video games is reliable for low-level perceptual and attentional skills but inconsistent for higher-order executive functions and memory (Bediou et al., 2018; Sala et al., 2018; Smith and Basak, 2023). A plausible explanation derives from the long-standing identical-elements view of transfer, which holds that gains acquired on one task generalize to another only to the extent that the two share specific stimuli, responses, or cognitive operations. Recent applications of this principle to the video-game training literature argue that transfer from action games is largely confined to perceptual and attentional processes that overlap with gameplay, with limited generalization to higher-order tasks lacking such overlap (Oei and Patterson, 2014). Two features of the present study likely reduced the power of the present design. First, the current study exposure threshold (≥30 min per day for the prior 6 months) is well below criteria used in studies that have reported gamer advantages (e.g., ≥11 h per week, Boot et al., 2011; lifetime cumulative gaming, Kühn and Gallinat, 2014); the present sample may simply not have accumulated the cumulative exposure that drives transfer. Second, transfer in the action-video-game literature depends on shared task demands between game and outcome (Bavelier and Green, 2025; Karle et al., 2010). PM tasks, used here, required maintaining intentions across delays and executing rule-governed retrieval procedures with no time pressure, sharing little with the perceptual–motor profile that gaming sharpens (Ihle et al., 2013). The present null should therefore be read as a boundary condition, not as evidence against gaming-cognition associations more broadly.

### 4.1 Limitations of the study

Several limitations should be noted. First, the PM tasks were not process-pure: each block emphasized one PM component while minimizing the other rather than crossing the two factorially, and the blocks also differed in incidental ways (e.g., time pressure, decision demands), so the “detection” and “retrieval” labels denote dominant processing demands rather than isolated components. Second, the two PM demands were administered in fixed-order blocks, which introduces potential order and strategy confounds; mixed-block, counterbalanced designs would provide a stronger test. Third, although each PM condition was based on a modest number of cue trials, the accuracy measures showed good internal consistency, so attenuation due to unreliability is unlikely to account for the weaker inhibition–retrieval association. Fourth, mixed data-collection modes (online vs. in-person) introduced measurement heterogeneity, though gaming effects remained null in both modes and after statistical control. Fifth, the gaming analysis used a modest exposure threshold and a cross-sectional design, limiting causal and transfer inferences. Finally, the sample was predominantly female (84.5%) and drawn from a single cultural context. Because gender is implicated in gaming behavior (Gisbert-Pérez et al., 2024; Phan et al., 2012), this may particularly constrain the gaming analyses, although gender was non-significant as a covariate (Supplementary Table S3). Replication in more gender-balanced and cross-cultural samples is warranted.

## 5 Conclusion

The present findings indicate that working memory and inhibitory control contribute differentially to prospective memory. Working memory was selectively associated with the detection phase, when an intention must be maintained and matched against incoming cues, while inhibitory control was associated with retrieval performance in the separate-model analysis without showing a significant phase-specific interaction. These results provide a within-subjects behavioral complement to recent neuroimaging and meta-analytic evidence that PM may be supported by partially separable mechanisms recruited as a function of phase demands (Cantarella et al., 2023; Peper and Hunter Ball, 2023), with the present data providing the strongest support for this view in the case of working memory. As a secondary observation, action video game experience at the exposure levels examined here was not associated with PM or its executive components; whether stricter exposure criteria or paradigms more closely matched to gaming demands would change this picture is a question for future work. Future studies might also examine shifting and sustained attention, which these tasks plausibly engage, as additional executive correlates of PM-analogue performance.

## Data Availability

The raw data supporting the conclusions of this article will be made available by the authors, without undue reservation.
