# Supplementary material for: Differential contributions of working memory and inhibitory control to prospective memory detection and retrieval
Source: Front Psychol. 2026 Aug 4;17:1871302. doi: 10.3389/fpsyg.2026.1871302 (PMC13481724; doi:10.3389/fpsyg.2026.1871302)
Supplement: Supplementary file 1 [file Data_Sheet_1.PDF]

## Supplementary Material

Table S1.
*Forward and backward digit span entered separately as predictors of PM detection and retrieval*

| Predictor | β | SE | *t* | *p* |
|---|---|---|---|---|
| *PM detection model* | | | | |
| Stroop interference | −.167 | .121 | −1.38 | .174 |
| Forward span (storage) | .115 | .124 | 0.93 | .355 |
| Backward span (executive) | .300 | .120 | 2.50 | .015 |
| Age | −.086 | .135 | −0.64 | .526 |
| Group (gamer) | −.070 | .269 | −0.26 | .795 |
| *PM retrieval model* | | | | |
| Stroop interference | −.273 | .124 | −2.21 | .031 |
| Forward span (storage) | .099 | .126 | 0.78 | .437 |
| Backward span (executive) | .042 | .123 | 0.34 | .734 |
| Age | .055 | .138 | 0.40 | .693 |
| Group (gamer) | −.301 | .275 | −1.09 | .278 |

*Note.* Detection $R^2 = .142$, $F(5, 64) = 2.11$, $p = .076$; Retrieval $R^2 = .103$, $F(5, 64) = 1.47$, $p = .211$. The detection–working-memory association is carried by backward (executive) span, not forward (storage) span.

Table S2.
*Storage versus executive components of working memory*

| **Predictor** | **β** | **SE** | ***T*** | ***p*** |
|---|---|---|---|---|
| *PM detection model* | | | | |
| Stroop interference | −.167 | .121 | −1.38 | .174 |
| Storage (forward span) | .159 | .122 | 1.31 | .196 |
| Executive (backward ∣ forward) | .300 | .120 | 2.50 | .015 |
| Age | −.086 | .135 | −0.64 | .526 |
| Group (gamer) | −.070 | .269 | −0.26 | .795 |
| *PM retrieval model* | | | | |
| Stroop interference | −.273 | .124 | −2.21 | .031 |
| Storage (forward span) | .105 | .124 | 0.84 | .403 |
| Executive (backward ∣ forward) | .042 | .123 | 0.34 | .734 |
| Age | .055 | .138 | 0.40 | .693 |
| Group (gamer) | −.301 | .275 | −1.09 | .278 |

*Note.* The executive component is backward span residualized on forward span. PM detection is predicted by the executive component, not the storage component; neither predicts PM retrieval. This converges with Table S1.

Table S3.
*Primary models with gender added as a covariate*

| **Predictor** | **β** | **SE** | ***t*** | ***P*** |
|---|---|---|---|---|
| *PM detection model* | | | | |
| Stroop interference | −.135 | .118 | −1.14 | .258 |
| Backward working memory | .320 | .120 | 2.66 | .010 |
| Age | −.067 | .136 | −0.49 | .627 |
| Group (gamer) | −.057 | .272 | −0.21 | .835 |
| Gender (female) | −.006 | .398 | −0.01 | .989 |
| *PM retrieval model* | | | | |
| Stroop interference | −.260 | .120 | −2.18 | .033 |
| Backward working memory | .047 | .122 | 0.39 | .700 |
| Age | .049 | .138 | 0.36 | .722 |
| Group (gamer) | −.316 | .276 | −1.14 | .257 |
| Gender (female) | −.358 | .404 | −0.89 | .378 |

*Note.* In the stacked cluster-robust model with gender added, the PM-component × working-memory interaction remained significant ($\beta = -.34$, $p = .010$) and gender was non-significant ($\beta = -.16$, $p = .587$). Maximum VIF = 1.38, indicating no collinearity.

Table S4.
*Primary models controlling for block-wise ongoing-task performance*

| **Predictor** | **β** | **SE** | ***t*** | ***p*** |
|---|---|---|---|---|
| *PM detection model* | | | | |
| Stroop interference | −.100 | .106 | −0.94 | .350 |
| Backward working memory | .300 | .108 | 2.78 | .007 |
| Age | −.140 | .123 | −1.14 | .257 |
| Group (gamer) | −.080 | .245 | −0.33 | .745 |
| Ongoing accuracy (detection block) | .407 | .108 | 3.78 | < .001 |
| *PM retrieval model* | | | | |
| Stroop interference | −.189 | .097 | −1.94 | .056 |
| Backward working memory | .033 | .099 | 0.34 | .737 |
| Age | .065 | .111 | 0.58 | .564 |
| Group (gamer) | −.321 | .225 | −1.43 | .158 |
| Ongoing accuracy (retrieval block) | .558 | .097 | 5.72 | < .001 |

*Note.* Detection $R^2 = .29$; Retrieval $R^2 = .40$. The detection–working-memory effect is unchanged; the retrieval–inhibition effect is attenuated to a non-significant trend ($p = .056$) after controlling for ongoing-task accuracy.

Table S5.
*Regression assumption diagnostics and robustness checks*

| Check | PM detection | PM retrieval | Judgement |
|---|---|---|---|
| Residual normality (Shapiro–Wilk) | W = .965, $p$ = .046 | W = .956, $p$ = .015 | Mild departure |
| Homoscedasticity (Breusch–Pagan) | $\chi^2(4) = 3.35$, $p$ = .501 | $\chi^2(4) = 2.83$, $p$ = .587 | Satisfied |
| Linearity (RESET) | $F$ = 1.91, $p$ = .156 | $F$ = 0.09, $p$ = .915 | Satisfied |
| Multicollinearity (max VIF) | 1.36 | 1.36 | Satisfied |

*Robustness of the focal effects to the mild non-normality:*

| Focal effect | OLS $p$ | HC3 robust $p$ | Bootstrap 95% CI (5,000) |
|---|---|---|---|
| Backward working memory → detection | .009 | .010 | [0.08, 0.54] |
| Stroop → retrieval | .042 | .044 | [−0.47, 0.00] |

*Note.* HC3 robust standard errors and a 5,000-replication case-resampling bootstrap both retain significance. The mild residual non-normality is attributable to the bounded accuracy scale.

Table S6.
*Equivalence check using the verbal forward-plus-backward composite*

| **Model / focal effect** | **β** | ***p*** |
|---|---|---|
| Detection — working memory (composite) | .32 | .009 |
| Detection — Stroop interference | −.20 | .097 |
| Retrieval — Stroop interference | −.27 | .030 |
| Retrieval — working memory (composite) | .11 | .384 |
| Stacked — PM-component × working memory | −.27 | .028 |

*Note.* The composite is the mean of z-standardized forward and backward span. The same pattern and conclusions hold, confirming that the choice of index does not drive the results.
